# A multilevel meta-analysis of the effects of repeated sprint training in hypoxia on athletic performance

**DOI:** 10.3389/fspor.2025.1641379

**Published:** 2025-08-21

**Authors:** Meng Han, Binglin Liu

**Affiliations:** 1 The College of Tianjin Renai, Tianjin, China; 2 College of Sports Training Science, Tianjin University of Sport, Tianjin, China

**Keywords:** repeated-sprint training, hypoxia, athletic performance, meta-analysis, repeated-sprint ability, aerobic capacity, anaerobic power, training adaptation

## Abstract

**Background:**

Repeated-sprint training in hypoxia (RSH) has emerged as a novel strategy to optimize repeated-sprint ability (RSA), aerobic capacity, and anaerobic performance in athletes. Although numerous studies have explored its efficacy compared with repeated-sprint training in normoxia (RSN), inconsistencies remain regarding overall benefits and modulating factors.

**Objectives:**

This study aimed to quantify the overall effect of RSH vs. RSN on athletic performance through a systematic review and multilevel meta-analysis and to identify categorical and continuous moderators influencing intervention efficacy.

**Methods:**

A comprehensive literature search was conducted across PubMed, Embase, Web of Science, and Cochrane Library up to January 31, 2025. Randomized controlled trials comparing RSH and RSN were included. Two reviewers independently screened, extracted, and assessed study quality. Random-effects multilevel models were used to calculate Hedges' *g* for overall and domain-specific outcomes (RSA, aerobic and anaerobic performance). Categorical (e.g., outcome types, intervention format, sex) and continuous moderators [e.g., inspired oxygen fraction (FiO_2_), intervention duration, frequency, and exercise-to-rest ratio] were tested via meta-regression. Publication bias was assessed through funnel plots and regression-based Egger tests.

**Results:**

Eighteen studies (*N* = 378 participants) were included, yielding 55 independent effect sizes. RSH significantly improved performance outcomes compared with RSN (*g* = 0.50, 95% CI: 0.34–0.67, *p* < 0.001). Subgroup analyses revealed stronger effects for RSA (*g* = 0.61) than for aerobic (*g* = 0.42) or anaerobic (*g* = 0.39) outcomes. Moderator analyses indicated that outcome type, exercise format, and FiO_2_) significantly moderated the effect size, with lower FiO_2_ (–13%–14%) and longer training duration (weeks) associated with greater gains. No sex differences were found. Funnel plot symmetry suggested low risk of publication bias.

**Conclusion:**

This meta-analysis confirms that RSH provides a moderate performance advantage over RSN, particularly for repeated-sprint ability. Specific implementation parameters such as moderate hypoxia intensity and sufficient training duration enhance efficacy. These findings offer evidence-based guidance for optimizing high-intensity interval training protocols under hypoxic conditions.

## 1 Introduction

Since the 1968 Mexico City Olympics first systematically demonstrated the regulatory potential of altitude environments on athletic performance, training strategies based on natural or simulated hypoxic conditions have emerged as key methods for optimizing competitive readiness in athletes (1). With the rapid expansion of high-altitude training centers worldwide—such as Iten in Kenya, St. Moritz in Switzerland, Haigeng in Kunming, and Duoba in Qinghai—hypoxic interventions have become deeply embedded within the periodized training frameworks of various sports  (1). The theory and practice of hypoxic training have evolved continuously. In the late 1980s, Soviet scholar Streltcov, drawing from clinical medicine, introduced the concept of intermittent hypoxic training (IHT) to improve cardiopulmonary function via periodic hypoxic exposure (2). Subsequently, Kaltchinskaia extended IHT into the athletic domain, proposing the adaptive hypothesis of “hypoxic stress–exercise unit activation”, marking a paradigm shift from rehabilitation to high-performance sports (3).

However, more than three decades of accumulated evidence have yielded mixed findings regarding the efficacy of IHT. Early studies suggested that hypoxic exposure could induce skeletal muscle angiogenesis and mitochondrial biogenesis via the Hypoxia-Inducible Factor 1 Alpha (HIF-1α) pathway (4). Yet, more rigorously controlled research later indicated that passive hypoxic stimulation alone fails to elicit these adaptations (5), unless combined with high- or maximal-intensity exercise capable of activating oxygen-sensitive signaling pathways (6, 7). A systematic review found that only 52.3% of studies involving intermittent training with hypoxia reported significant performance benefits (8), while another meta-analysis on Live Low–Train High (LLTH) models showed no statistical advantage over conventional training in enhancing sea-level endurance performance (9). This inconsistency has been attributed to the intensity-dependent nature of LLTH: only when the training intensity in hypoxia exceeds the individual anaerobic threshold can it effectively overcome the oxygen transport bottleneck to improve performance (10). In response, researchers have sought to develop novel paradigms that transcend the limitations of traditional IHT. One such strategy, repeated-sprint training in hypoxia (RSH), was proposed by Faiss in 2013. RSH combines maximal-effort sprint bouts (≤30 s) with moderate hypoxic exposure (FiO_2_ = 14.5%–16.4%, simulating 2,000–3,000 meters altitude), thereby creating a dual stimulus of high intensity and low oxygen availability (11). The physiological advantages of RSH span three domains: (1) at the molecular level, it activates glycolytic gene expression via the AMPK/PGC-1α pathway (7); (2) at the microvascular level, it enhances muscle oxygen uptake and reperfusion kinetics (12); and (3) at the neuromuscular level, it improves type II fiber recruitment thresholds and phosphocreatine buffering capacity (13). Notably, RSH protocols maintain an incomplete recovery state by enforcing short exercise-to-rest ratios (<1:6), thereby sustaining metabolic stress and avoiding the attenuation of adaptive responses observed in traditional IHT due to excessive recovery (3).

Evidence suggests that RSH can significantly enhance repeat sprint ability (RSA) and peak anaerobic power in elite athletes (14). Moreover, since its inception, RSH has shown expanding physiological benefits, including improved muscle oxygen diffusion capacity and lactate clearance rates (11), as well as superior performance gains compared to normoxic repeated-sprint training (RSN) in team sports settings (15). Nonetheless, existing studies vary widely in terms of participant populations (e.g., adolescents vs. elite athletes), hypoxic delivery modes (e.g., terrestrial vs. simulated), training protocols (e.g., intensity, duration), and outcome measures (e.g., strength, speed, cardiorespiratory fitness). Such heterogeneity has led to ongoing debate regarding the true efficacy of RSH, with findings ranging from significant improvements to non-significant effects or even detrimental trends. These inconsistencies may be influenced by complex factors including research design, nested outcome structures, and statistical non-independence among data sources.

To address these challenges, the present study adopts a multilevel meta-analytic framework, which overcomes the limitations of traditional meta-analyses that treat each study as contributing a single effect size. By modeling nested data structures, this approach accounts for statistical dependencies among multiple outcomes, time points, or groups within a single study, thereby enhancing the robustness and interpretability of the results. Furthermore, to comprehensively characterize the impact of RSH on athletic performance, we incorporated both categorical and continuous moderators—such as training mode, oxygen concentration, and intervention duration—into a multivariate meta-regression model. This allows us to explore key determinants and boundary conditions shaping the effectiveness of RSH.

## 2 Methods

### 2.1 Literature search strategy

This meta-analysis was conducted in accordance with the PRISMA 2020 (Preferred Reporting Items for Systematic Reviews and Meta-Analyses) guidelines. A systematic search of five major databases—PubMed, Web of Science, Embase, Scopus, and ScienceDirect—was conducted up to 31 January 2025. Boolean logic was applied to construct the search strategy, using the following terms: “repeated sprint” OR “repeated sprint” OR “sprint interval” AND “hypoxia” OR “low oxygen” OR “altitude” AND “performance” OR “athletic performance” OR “endurance” OR “power” OR “VO_2max_”. No restrictions were placed on language or publication date to ensure comprehensiveness. In addition, the reference lists of all eligible articles and relevant reviews were manually screened to identify any potentially missing studies. All search results were managed using EndNote 20, and duplicates were removed using the built-in “Find Duplicates” function.

### 2.2 Eligibility criteria

Study screening was performed independently by two reviewers (MH and BL) in two distinct stages, starting with title and abstract screening, followed by full-text evaluation. Studies were eligible for inclusion if they met the following criteria: they employed a randomized controlled trial (RCT) design with parallel-group allocation; participants were trained individuals, athletes, or adults with at least moderate levels of physical activity; the intervention group received RSH; the control group performed the same RSN; at least one quantifiable performance outcome was reported, such as sprint time, peak power, RSA, maximal oxygen uptake (VO_2max_), cardiorespiratory function, or Wingate test results; and sufficient data were available to calculate Hedges' *g*, including pre- and post-intervention means, standard deviations, and sample sizes, or equivalent statistics. Studies were excluded if they were not randomized, were published as abstracts, reviews, or animal studies, involved combined interventions in which RSH effects could not be clearly isolated, or did not provide sufficient data and failed to respond to requests for additional information. Any disagreements between reviewers were resolved through discussion with a third researcher.

### 2.3 Risk of bias assessment

The risk of bias in the included studies was assessed using the Cochrane Risk of Bias 2.0 tool (RoB 2.0), as recommended by the Cochrane Collaboration (16). This instrument evaluates five domains of potential bias: the randomization process, deviations from intended interventions, missing outcome data, measurement of the outcome, and selection of the reported result. Each domain was rated as “low risk,” “some concerns,” or “high risk,” and an overall risk-of-bias judgment was derived accordingly. Two reviewers (ML and DL) independently performed the assessment, and any discrepancies were resolved through discussion with a third reviewer.

### 2.4 Data extraction

A standardized data extraction form was developed prior to analysis. Extracted data included: first author, year of publication, country/region, study design, intervention duration and frequency, inspired oxygen fraction (Fio_2_), training modality, sample size, participant characteristics, outcome type, and pre- and post-intervention means and standard deviations. Data were independently extracted by two reviewers (ML and DL) and cross-checked upon completion. For studies lacking numerical data, outcomes were extracted from figures using GraphDigitizer (17) or obtained directly from the corresponding authors. All extracted data were entered into a structured Excel spreadsheet, and consistency was verified through cross-validation in R.

### 2.5 Data synthesis

Effect sizes were calculated using Hedges' *g*, representing the standardized mean difference between pre- and post-intervention changes in the experimental and control groups. This metric accounts for small sample bias and is widely recommended for continuous outcome measures in meta-analyses. In the present study, Hedges' *g* was computed based on the difference in mean change scores between groups, following the formula below (18, 19):

$$g=j\cdot \frac{{\left(M_{\mathrm{post}}-M_{\mathrm{pre}}\right)}_{\mathrm{RSH}}-{\left(M_{\mathrm{post}}-M_{\mathrm{pre}}\right)}_{\mathrm{RSH}}}{SD_{\mathrm{pooled}}}$$

The correction factor $J=1-\frac{3}{4(df)-1}$, was used to adjust for small sample bias. The pooled standard deviation was calculated using the following formula:

$$SD_{\mathrm{pooled}}=\sqrt{\frac{(n_{1}-1)SD_{1}^{2}+(n_{2}-1)SD_{2}^{2}}{n_{1}+n_{2}-2}}$$

Variance estimation followed the procedures described by Borenstein et al. (20, 21). When the standard deviation of the change score was not reported, it was estimated using the following formula, assuming a pre–post correlation of *r* = 0.5 (22):

$$SD_{\Delta }=\sqrt{SD_{\mathrm{pre}}^{2}+SD_{\mathrm{post}}^{2}-2r\cdot SD_{\mathrm{pre}}\cdot SD_{\mathrm{post}}}$$

Hedges' *g* and its corresponding variance (var_g) were ultimately used for meta-analytic modeling.

### 2.6 Statistical analysis

A multilevel random-effects model was employed to perform the meta-analysis, aiming to address the statistical non-independence introduced by multiple effect sizes within individual studies, such as those arising from different outcome measures or time points. The model was constructed as a two-level nested structure (study/outcome), where the observed effect size was decomposed into the overall mean effect, a study-level random effect, an outcome-level random effect, and a residual sampling error based on the known variance of each effect size. Model fitting was performed using the rma.mv() function in the metafor package in R, with parameters estimated via restricted maximum likelihood (REML). Multilevel heterogeneity indices (*I*²) were calculated using the i2_ml() function, allowing for decomposition into between-study and within-study components. To explore potential sources of heterogeneity, a series of multivariate meta-regression models were constructed, incorporating both continuous (e.g., FiO_2_ concentration, training duration, frequency) and categorical moderators (e.g., training modality, outcome type). Moderator variables were coded according to their measurement scale, with categorical variables dummy-coded accordingly. All models retained the random-effects structure to account for the nesting of effect sizes within studies. Meta-regression results are reported as regression coefficients, standard errors, 95% confidence intervals, and *p*-values. Model fit was evaluated using Akaike Information Criterion (AIC) and marginal R^2^. For continuous moderators, moderation effects were visualized using the bubble_plot() function from the orchard package, with bubble size proportional to effect size precision (1/SE). Publication bias was assessed by constructing enhanced funnel plots via the viz_funnel() function from the metaviz package (23), alongside Egger's regression test to detect small-study effects. In cases where the primary model was multilevel and the regtest() function could not be applied, linear model approximations were used instead. When publication bias was evident, the trim-and-fill method was used to adjust the overall effect size (24).

## 3 Results

### 3.1 Study selection and inclusion

The systematic search and screening process was conducted in accordance with the PRISMA 2020 guidelines (25). A total of 478 records were initially identified. After removing duplicates using EndNote 20, 263 articles remained for title and abstract screening. During this stage, 216 studies were excluded based on the following criteria: non-athlete populations, non-interventional designs, interventions not aligned with the study objectives (e.g., absence of repeated-sprint training or lack of hypoxic exposure), or the absence of a control group. The remaining 47 articles were subjected to full-text review based on pre-specified inclusion criteria. Studies were eligible if they met the following conditions: (1) a randomized controlled trial (RCT) with a parallel-group design; (2) the intervention group performed repeated-sprint training under hypoxic conditions with inspired oxygen fraction (FiO_2_) below normoxic levels (<20.9%); (3) the control group underwent the same training protocol under normoxic conditions (RSN); (4) participants were healthy trained individuals or competitive athletes; (5) at least one quantifiable performance-related outcome was reported, such as RSA, VO_2max_, or PPO; and (6) sufficient data were available (i.e., pre- and post-intervention means, standard deviations, and sample sizes), or could be obtained from the authors upon request. Articles were excluded if they involved non-RSH interventions, inappropriate study designs, or lacked sufficient data for effect size estimation.

**Figure 1:**
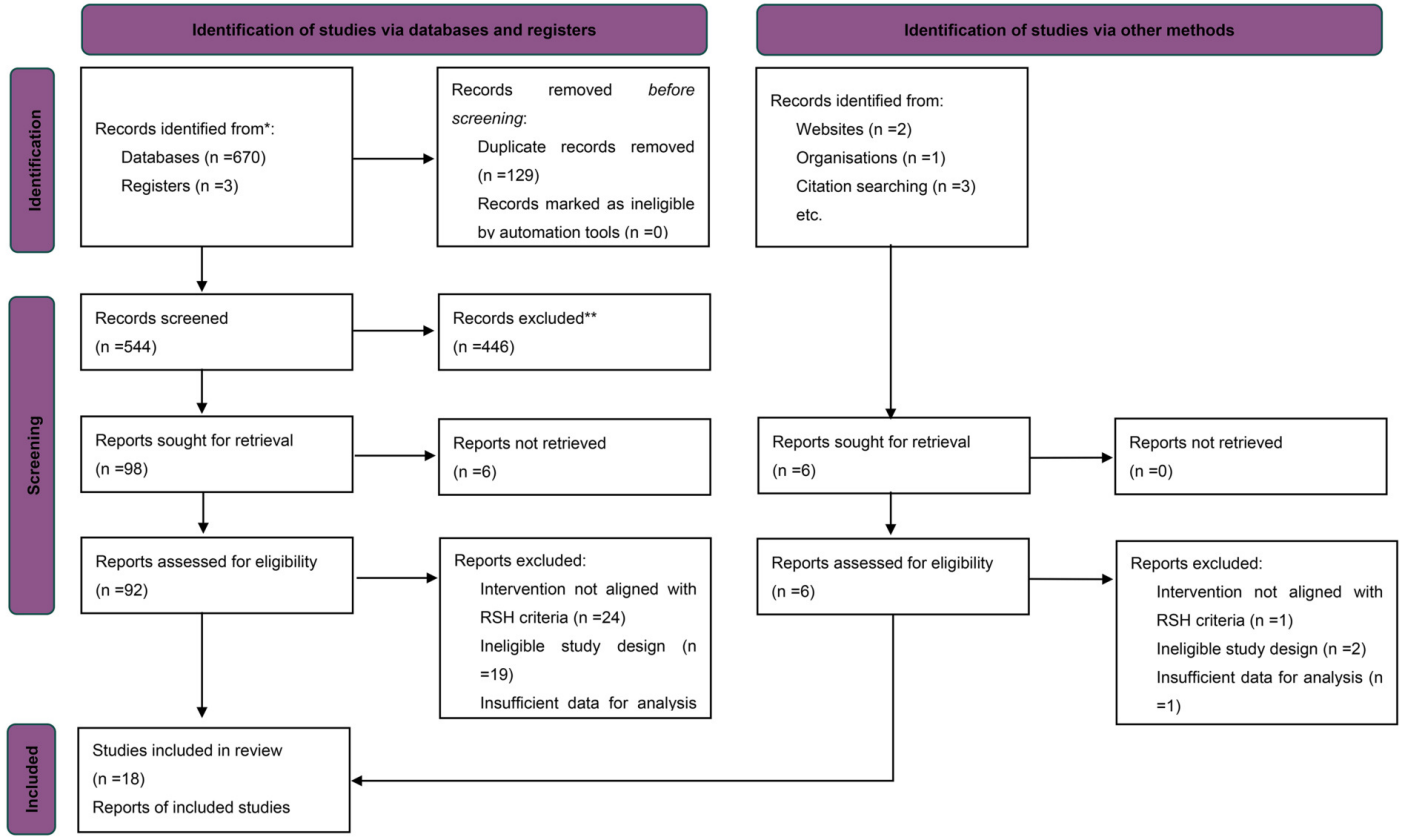
PRISMA flow diagram of study selection.

In total, 18 studies published between 2013 and 2025 met the inclusion criteria. All were published in English and collectively included 386 athlete participants. The study selection process is summarized in the PRISMA flow diagram (Figure 1). Overall, the screening ensured that included studies were of high methodological quality, exhibited acceptable homogeneity, and provided sufficient extractable data to support the subsequent multilevel meta-analysis.

### 3.2 Study characteristics

A total of 18 randomized controlled trials published between 2013 and 2024 were included in the analysis (11, 26–42). These studies were conducted across various countries and regions in Europe, Asia, and Oceania, and all involved adolescent or adult competitive athletes participating in sports such as football, rugby, tennis, and sprinting. The cumulative sample size was 370 participants, with ages predominantly ranging from 15–30 years. All studies included both a RSH group (FiO_2_ = 13.0%–14.8%) and RSN group (FiO_2_ = 20.9%). See in Table 1.

**Table 1:** Summary of study characteristics of included randomized controlled trials.

Author, Year	Type	Subjects	Group	Duration (weeks)	Frequency (times)	Repeated Sprint Training Parameters	Outcome Measures	Main conclusions
Faiss et al. (11)	RCTs	50 male cyclists with moderate training level, 35 ± 7 years old, 179 ± 5 cm, 75 ± 9kg	RSH (*n* = 20, FiO_2_ = 14.6%) vs RSN (*n* = 20, FiO_2_ = 20.9%) vs control group (*n* = 10)	4	2	3 sets × 5 10s all-out sprints (1:2 work-rest ratio), 5 min rest between sets	RSA performance (sprint count, power output), NIRS hemodynamics, muscle mRNA expression, enzyme activity, electrophysiology (EMG);	RSH significantly increased the RSA sprint number and blood perfusion changes, induced upregulation of HIF-1*α*, CA3, and MCT-4 expression, and downregulation of TFAM and PGC-1α, suggesting a bias toward glycolytic adaptation, and RSH was superior to RSN
Galvin et al. (34)	RCTs	30 elite rugby academy players, 18.4 ± 1.5 years old, 183.0 ± 6.6 cm, 88.1 ± 8.9 kg	RSH (*n* = 15, FiO_2_ = 13%) vs RSN (*n* = 15, FiO_2_ = 21%)	4	3	10 times × 6 s sprint, rest 30 s (treadmill), a total of 1 set	Yo-Yo IR test, m-sprint, RSA test (speed, distance, descent rate), NIRS, VO, VCO, SaO, HR, [La];	Yo-Yo performance in the RSH group improved by 33%, which was higher than that in the RSN group (14%); brain deoxygenation was reduced and oxygen consumption increased, indicating oxidative metabolic adaptation; there was no significant difference in sprint speed, which is suitable for enhancing aerobic interval performance
Gatterer et al. (35)	RCTs	16 elite adolescent male soccer players, 15.3 ± 0.5 years old, 173 ± 7 cm, 62.6 ± 6.6 kg	RSH (FiO_2_ = 14.8%, *n* = 8) vs RSN (FiO_2_ = 20.9%, *n* = 8)	5	2	3 sets × 5 times × 10s radial shuttle run (4.5 m), 20s rest, 5 min between sets	YYIR running distance, RSA (average time, optimal time, % fatigue rate, fatigue slope), [La];	The RSH group significantly improved the RSA fatigue slope (*p* = 0.024), and there was no significant difference in YYIR performance; the training is feasible and suitable for small hypoxic chambers, suggesting that it improves the speed maintenance ability
Brocherie et al. (30)	RCTs	16 high-level U18 male football players, male, average age 17.1	RSH group (*n* = 8, FiO_2_ = 14.3%) vs RSN group (*n* = 8, FiO_2_ = 21.0%)	5	2	High-intensity interval running (90%–110% MAS), 15s work/15s rest, combined with agility training, jumping and other explosive tasks	CMJ height, -m sprint time, MSS (MSS), MAS (MAS), repeated sprint performance (RSAbest, RSAmean, RSAsdec), agility ability (RAbest, RAmean, RAsdec);	Both RSH and RSN improved explosive power and repeated sprinting ability (*p* < 0.05), but only RSH significantly improved agility (RAbest and RAmean); RSH training is more effective for neuromuscular adaptation and may be more suitable for improving direction change ability.
Brocherie et al. (31)	RCTs	Elite male hockey players, *N* = 32, age 25.3 ± 4.6 years	RSH,RSN	2	3	4 sets×5 5-second sprints, 25-second rest, 5-minute rest between sets	Hbmass (CO reabsorption method), YYIR (high-intensity interval running), RSA test × m);	Both LHTLH and LHTL significantly increased Hbmass (+3–4%), with the LHTLH group experiencing a greater increase in RSA, with the effect lasting for 3 weeks; YYIR2 increased in both groups, with no significant change in jumping ability.
Faiss et al. (33)	RCTs	Swiss national cross-country skiing team athletes, *N* = 17 (11 males, 6 females), average age 27	RSH group (FiO_2_ = 13.8%, *n* = 9) vs. RSN group (FiO_2_ = 20.9%, *n* = 8)	2	3	4 sets × 5 times × 10 s parallel bar skiing simulation sprint, 20 s interval, 3 min rest between sets	Sprint power output (last time, th time), NIRS muscle oxygenation, electromyography (EMG), blood [La], SpO₂, RPE;	The RSH group showed a significant improvement in the final sprint ability (5th sprint power ↑), fast reperfusion, large muscle deoxygenation, enhanced EMG, and higher oxygen utilization efficiency than RSN, which is suitable for improving the final explosive power of skiing events.
Kasai et al. (38)	RCTs	32 college women's lacrosse players, approximately 20 years old, born and trained at sea level	RSH group (FiO_2_ = 14.5%, *n* = 16) vs RSN group (FiO_2_ = 20.9%, *n* = 16)	4	2	2 sets × 10 times × 7 s bicycle sprint, 30 s rest, 20→10 min rest between sets, load is 4% of body weight	performance: PPO, MPO, FI; physiological indicators: VO2max, TTE (Time to Exhaustion), SpO₂;	The RSH group had a significant increase in average power in all sprints, peak power increased 3 times that of the RSN group, TTE was significantly prolonged, and VO2max remained unchanged; the results support that RSH can significantly enhance the maximum anaerobic output and repeated sprint capacity of female team athletes
Hamlin et al. (37)	RCTs	19 New Zealand amateur male rugby players (HYP group *n* = 9, NORM group *n* = 10), average age ∼21 years, weight about 77–88 kg, height ∼174–178 cm	HYP group (FiO_2_ = 14.5%) vs NORM group (FiO_2_ = 20.9%)	1	2	Each training session consisted of 4 sets of 5 5-second sprints, 5 min of rest between sets, 25 s of rest within sets, and all-out sprints on the Wattbike.	repeated sprint fatigue index (two algorithms); Yo-Yo Level recovery test (YYIR;	The HYP group had significantly lower sprint fatigue than the NORM group in the 2nd to 5th test after intervention, and performance continued to improve. YYIR1 performance improved in both groups, with no significant difference between the groups. Supplementary intervention had limited effect on further improvement in any group. The HYP group had a higher RPE and greater training stress, suggesting that training load was part of the source of the effect.
Brechbuhl et al. (28)	RCTs	20 tennis players (16 males, 4 females); age approximately 23.8 years	RSH group (*n* = 9, FiO_2_ = 14.5%) vs RSN group (*n* = 9, FiO_2_ = 20.9%)	2	3	4 sets × 5 shuttle runs (6s sprint/24s rest recovery)	Tennis-specific extreme endurance test (TEST) performance (TTE, time to OBLA, time to VT; tennis technical performance: Ball velocity (BV), accuracy (BA), TP; RSA performance (RSAbest, RSATT, Sprint decrement); physiological indicators [VO2max, VEmax, (La)max, HRmax];	The RSH group significantly improved TTE (+14.6%), time to OBLA (+40.1%), and VT2 (+23.6%), and BA (+13.8%) and TP (+13.8%) also improved significantly; the RSN group did not show significant improvement; there was no difference in RSA indicators between the groups, but RSH showed better technical maintenance ability.
Beard et al. (26)	RCTs	International rugby players (RSH group *n* = 10, RSN group *n* = 9), average age 23.0–25.6 years	RSH (FiO_2_ = 13.8%, 3,000 m) vs RSN (FiO_2_ = 20.9%, 300 m)	2	2	Each training session: 3 sets × 8 reps×10-second bicycle sprints (20 sec rest, 2 min rest between sets), total hypoxic exposure time 96 min	RSA ② Peak power (PPO), average power (MPO), power attenuation rate (Sprint decrement)	The PPO in the RSH group was significantly improved (*P* < 0.01), and the MPO was significantly increased (*P* < 0.001), while there was no significant change in the RSN group. Short-term 4-session RSH can effectively enhance the repetitive power output of the lower limbs of international-level rugby players, which has practical competitive significance.
Beard et al. (27)	RCTs	World-class international rugby players, 36 (RSH = 18, RS*N* = 18), male, average age 24.1 ± 2.7 years	RSH group: FiO_2_ = 13.8%, simulated 3,000 m; RSN group: FiO_2_ = 20.9%, normoxia	2	2	3 sets × 8 reps × 10 s sprint, 20s rest, 2 min rest between sets, using a bipolar rowing machine	PPO (Peak Power), MPO (Mean Power), Sdec (Sprint Decay)	In the RSH group, PPO increased significantly (+42 W, *p* = 0.002) and MPO increased significantly (+38 W, *p* < 0.001), while there was no significant change in the RSN group. Upper limb RSH has a positive effect on improving RSA of international rugby players and is suitable for short-term preparation period.
Kasai et al. (39)	RCTs	18 male university sprint runners (100–200 m), age 20.0 ± 0.3 years, height 175.9 ± 1.1 cm, weight 65.0 ± 1.2 kg, training years 8.1 ± 0.5 years	HYPO group (FiO_2_ = 14.5%, *n* = 9) vs NOR group (FiO_2_ = 20.9%, *n* = 9)	1	6	Daily training includes 15s, 30s and 6s sprint intervals at 6%–7% body weight; includes 3 training modes, SpO₂ control, and recovery intervals of 5–10 min	RSA test (10 × 6s sprint), 30s maximum power, VO2max, 60 m run (including 0–10 m analysis), muscle PCr content (31P-MRS)	RSH did not significantly improve RS performance or 60 m time, but significantly improved 0–10 m sprint performance and muscle PCr content, indicating its potential benefit for explosive power output
Camacho-Cardenosa et al. (32)	RCTs	24 male college athletes, average age 22.7 ± 2.9 years, weight 70.2 ± 3.4 kg	RSH group (*n* = 8, FiO_2_ = 14.5%) vs RSN group (*n* = 8, FiO_2_ = 20.9%) vs CON group (*n* = 8)	4	2	Each training session included 2 sets of 5 10-second all-out sprints, 10 min of rest between sets, 20 s of rest between sprints, and a constant power of 120W.	Anaerobic capacity: Wingate test (peak power, average power, lactate, maximum heart rate)	The maximum power and sprint times in the RSH group were significantly improved (+14.96%, +20.36%, *p* < 0.05), with medium to large effect sizes (ES ≈ 0.71–0.78); VO2max and jumping ability did not change significantly, indicating that RSH can be used to improve anaerobic and sprint capacity in the short term.
							RSA: sprint times (10-second all-out cycling test)	
							Aerobic capacity: Interval recovery test (Yo-Yo IR)	
							Lower limb explosive power: SJ and CMJ	
Brechbuhl et al. (29)	RCTs	Elite tennis players at ITN level 1–2 (RSH = 11, RSN = 11, CON = 8), aged 18–35 years	RSH group (FiO_2_ = 14.5%) vs RSN group (FiO_2_ = 20.9%) vs CON group (conventional training)	2	2	Each training session consisted of 4 sets of 4 maximum sprints (∼8 s), 22 s of recovery between sprints, 5 min of recovery between sets, combined with a tennis hitting task	RSA test (RSAbest, RSATT)	In the RSH group, TTE and OBLA times were significantly improved (+18.3%, +24.4%), RSA performance was improved, TPmax increased by 46.3%, and blood perfusion was enhanced, while similar changes were not observed in the RSN and CON groups, and there was no significant difference in HRV.
							TEST special endurance test (TTE, OBLA point)	
							③ Batting performance (ball speed BV, accuracy BA, comprehensive TP)	
							Hemodynamics (NIRS: tHb, HHb)	
							HRV (RMSSD, LF, HF)	
Pramkratok et al. (40)	RCTs	Thai national rugby sevens players, *N* = 14, male, average age about 24 years old	RSH group (FiO_2_ = 14.5%, *n* = 7); RSN group (normoxia, FiO_2_ = 20.9%, *n* = 7)	6	3	3 sets of 10 reps of 6-second treadmill sprints (140% vVO2peak), 30-second passive recovery between sprints, 4 min between sets	VO2peak, extreme exercise time, fatigue index (FI), muscle oxygenation level (TSI, O2Hb, HHb, tHb), hemoglobin, hematocrit, HIF-1α, VEGF	The VO2peak, extreme exercise time, and fatigue index of the RSH group were significantly improved, the muscle deoxygenation capacity was enhanced, and the serum HIF-1α and VEGF concentrations increased, indicating that muscle angiogenesis is involved in training adaptation
Giovanna et al. (36)	RCTs	Endurance-trained male athletes, *N* = 39, age 25.6 ± 5.7 years	HG (hypoxia group, FiO_2_ ≈ 13%, *N* = 10); BFRG (blood blockage group during exercise, *N* = 10); BFRrG (blood blockage group during recovery, *N* = 10); CG (normoxic control group, *N* = 9)	2	3	4 sets × 5 times × 10 s of full-force cycling, 5–10 min of recovery between sets, 20 s of active recovery between sprints, load 0.8 Nm/kg	Peak aerobic power, maximum cumulative oxygen uptake, time trial performance, force-speed relationship parameters, anaerobic capacity (maximum oxygen debt), respiratory exchange rate	Two weeks of RST can improve peak aerobic power, maximum cumulative oxygen uptake and maximum oxygen debt, but anaerobic and hypoxic interventions did not bring additional benefits, and time trials and muscle mechanical properties did not show significant improvements
Shi et al. (41)	RCTs	College male football/handball players, *N* = 32, average age about 20 years old	RSH2-wk (2-week hypoxia group, *n* = 10); CON2-wk (2-week normoxic control group, *n* = 12)	5	3	3 sets × 5 times × 5 s of full sprint, 25 s of passive recovery, 5 min of passive recovery between sets, 20% of TRmax of treadmill resistance	Yo–Yo IR1 results, VO2max, maximal sprint speed, 4-set repeated sprint performance in a team sport simulation protocol (peak/mean speed, horizontal force, power), total work, blood lactate, s-RPE, heart rate	Hypoxic RST (RSH2-wk and RSH5-wk) both significantly improved RSA in a team sport simulation protocol, more effectively than normoxic training. 5 weeks of RSH can maintain training benefits for a longer period of time and is recommended for pre-season preparation.
Thongsawang et al. (42)	RCTs	Female college football players, *N* = 39, average age about 20.5 years old	RSH (*n* = 11); RSN (*n* = 9)	4	3	3 sets × 6 times × 6 s sprint (140% vVO2max), 30 s passive recovery between sprints, 5 min between sets, 1% slope, treadmill	Maximum oxygen uptake, maximum speed, exercise tolerance time, repeated sprint ability (peak power, average power, fatigue index, lactate), blood oxygen saturation, hemoglobin, hematocrit, red blood cell count, platelet count	The RSH + cordyceps group significantly improved TTE, average power, lactate level, and fatigue index, but did not improve VO2max; there was no significant change in hematological indicators, suggesting that cordyceps may enhance exercise performance through non-hematopoietic mechanisms

Abbreviations: RSH, repeated sprint training in hypoxia; RSN, repeated sprint training in normoxia; FiO2, fraction of inspired oxygen; RSA, repeated sprint ability; m-sprint, maximal sprint; NIRS, near-infrared spectroscopy; EMG, electromyography; HIF-1α, hypoxia-inducible factor 1 alpha; CA3, carbonic anhydrase III; MCT-4, monocarboxylate transporter 4; PGC-1α, peroxisome proliferator-activated receptor gamma coactivator 1 alpha; YYIR, Yo-Yo intermittent recovery test; PPO, peak power output; MPO, mean power output; TTE, time to exhaustion; VO2max, maximal oxygen uptake; VCO2, carbon dioxide output; SaO2, arterial oxygen saturation; HR, heart rate; [La], blood lactate concentration; BFR, blood flow restriction; OBLA, onset of blood lactate accumulation; RPE, rating of perceived exertion; VEmax, maximal ventilation; PCr, phosphocreatine; vVO2peak, velocity at maximal oxygen uptake; MSS, maximal sprint speed; MAS, maximal aerobic speed.

Intervention durations ranged from 1–6 weeks, with training frequencies typically set at 2–3 sessions per week. The repeated-sprint protocols were implemented in the form of running, cycling, or sport-specific exercises. A typical training session consisted of 2–6 sets, each comprising 4–10 sprints lasting 5–10 s, with 20–30 s of intra-set rest and 2–5 min of inter-set rest. Primary outcome measures included repeat sprint ability (e.g., RSAbest, RSATT), peak and mean power output (PPO, MPO), maximal oxygen uptake (VO_2max_), explosive performance (e.g., CMJ, SJ), and aerobic intermittent capacity (Yo-Yo IR1/IR2). Several studies also assessed physiological mechanisms such as muscle oxygenation, heart rate variability, and blood biomarkers, providing a robust dataset for subsequent meta-analytic modeling.

### 3.3 Risk of bias assessment in included studies

**Figure 2:**
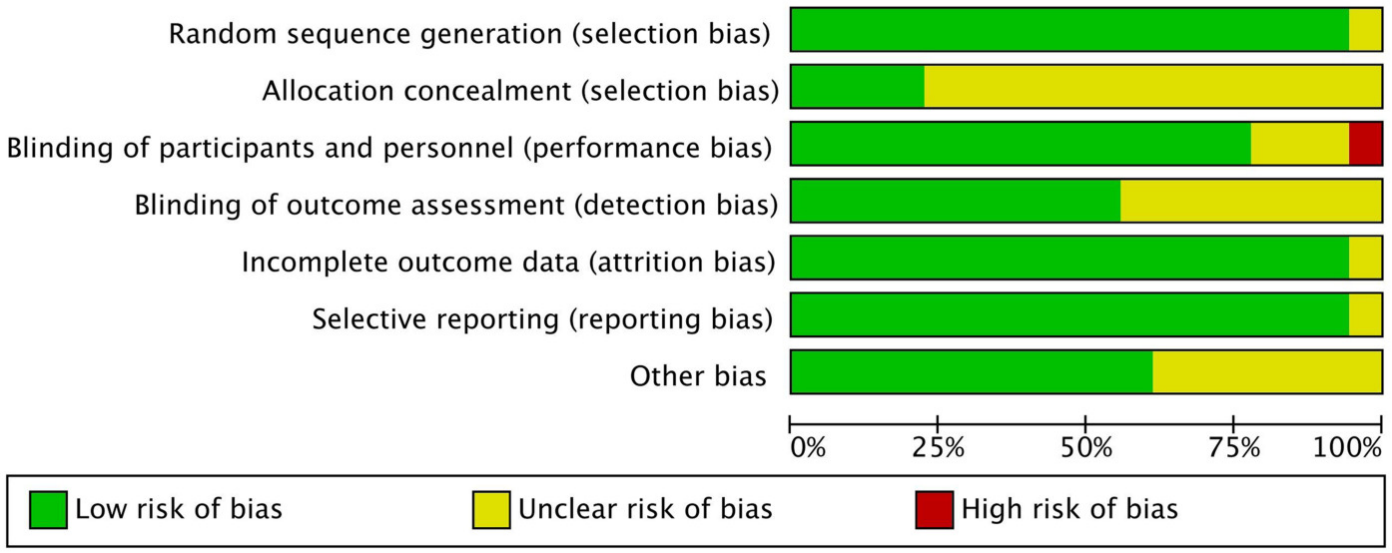
Risk of bias assessment across included studies.

**Figure 3:**
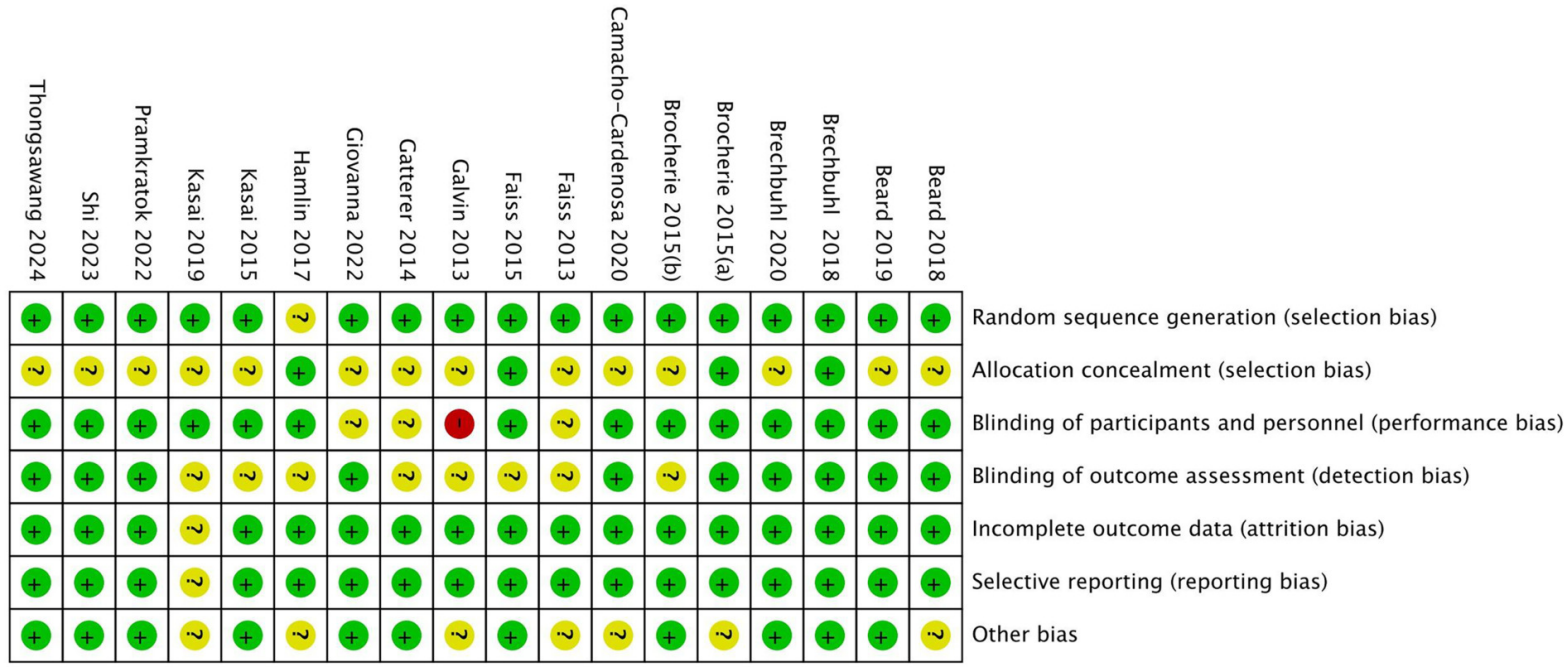
Overall risk of bias across all included studies.

In the quality assessment of the 18 included studies, most studies showed a low risk of bias for random sequence generation (selection bias), allocation concealment (selection bias), blinding of outcome assessment (detection bias), and selective reporting (reporting bias), as indicated by the green marks. However, it is important to note that due to the nature of the intervention-based RCTs, achieving full blinding of participants and personnel (performance bias) is often not feasible. Consequently, this aspect of the risk of bias is challenging to control, with some studies showing unclear (yellow marks) or high risk (red marks) in this domain. Additionally, some studies presented unclear risks regarding incomplete outcome data (attrition bias). Nevertheless, the majority of studies demonstrated high quality, with no significant evidence of systematic bias. Future research should aim to ensure stringent blinding where possible to further minimize potential bias. See Figures 2, 3.

### 3.4 Overall effect estimates (main effects from multilevel modeling)

A total of 53 independent effect sizes were synthesized using a multilevel random-effects model. The results indicated that RSH produced a statistically significant advantage over RSN in enhancing athletic performance, with a pooled moderate effect size [Hedges' *g* = 0.35, 95% CI (0.18, 0.51), *p* < 0.01]. The between-study heterogeneity was low (*I*^2^ = 24.83%), suggesting good consistency across studies. The prediction interval (PI) ranged from −0.16–0.85, indicating that while future studies may observe varying effects, the overall trend remains favorable toward RSH.

Among the pre-defined outcome categories, RSH showed the most pronounced effect on RSA, with a moderate-to-large pooled effect size [ES = 0.50, 95% CI (0.12, 0.88), *p* < 0.01] and moderate heterogeneity (*I*² = 34.28%). Although the prediction interval was wide [PI = (–0.75, 1.75)], implying possible nonsignificant or even negative effects under certain conditions, the overall pattern supports the beneficial impact of RSH on RSA outcomes.

In terms of aerobic capacity, RSH also demonstrated a statistically significant benefit compared with RSN [ES = 0.23, 95% CI (0.02, 0.44), *p* = 0.03], with very low heterogeneity (*I*² = 3.52%), indicating a high degree of consistency among studies. The prediction interval (–0.04, 0.49) suggests that while some future findings may cluster near null effects, the general tendency favors a positive outcome.

**Figure 4:**
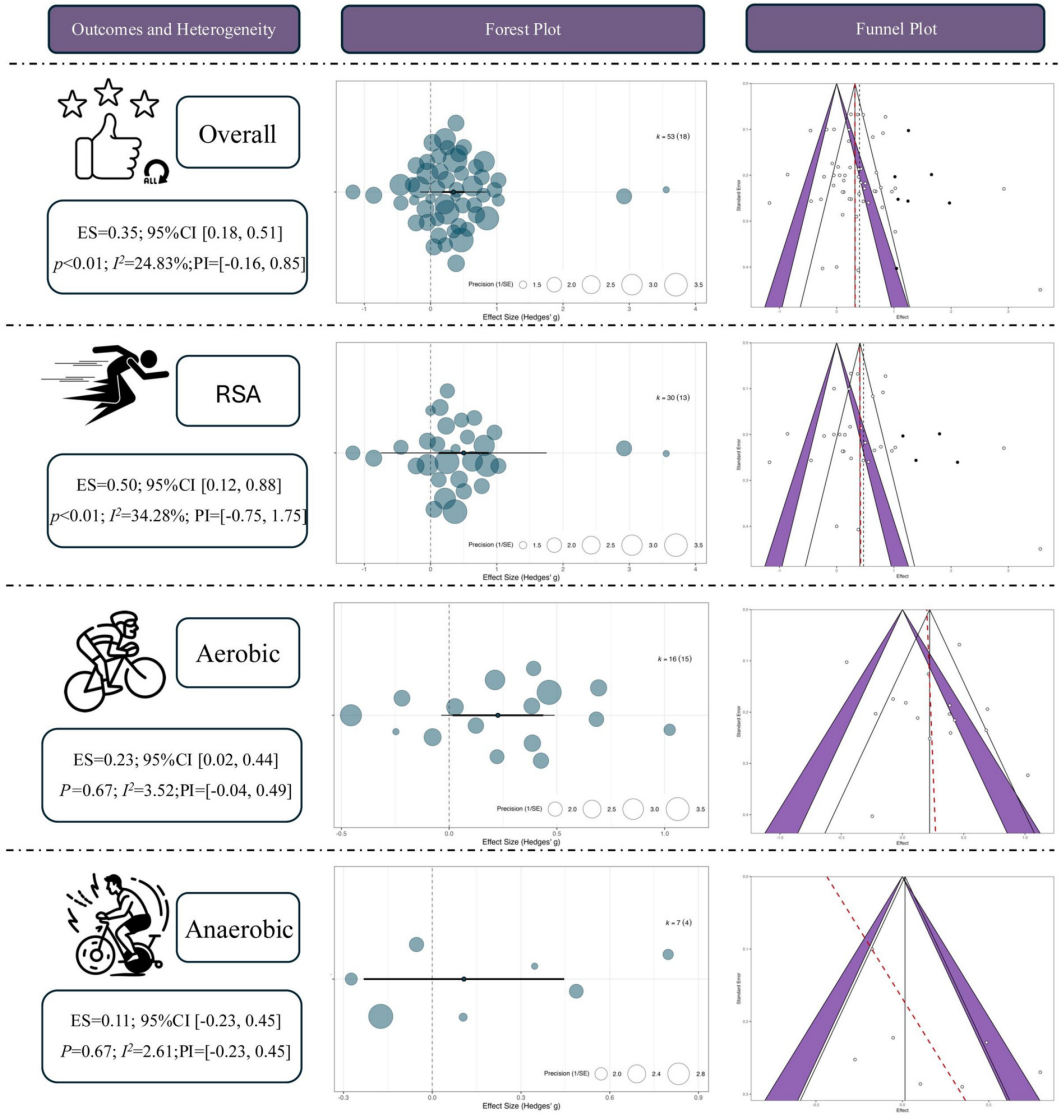
Overall effect of repeated sprint training in hypoxia (RSH) on athletic performance. ES, effect size; CI, confidence interval; RSA, repeated sprint ability; P, *p*-value; *I*², I-squared (heterogeneity statistic).

In contrast, the evidence supporting an improvement in anaerobic performance was not statistically significant [ES = 0.11, 95% CI (–0.23, 0.45), *p* = 0.67], and heterogeneity was minimal (*I*² = 2.61%). The prediction interval (–0.23, 0.45) reflects a lack of consistency in observed effects, which may be attributed to variation in sample sizes, intervention durations, or measurement methods across studies. See in Figure 4.

### 3.5 Moderator analysis

#### 3.5.1 Categorical moderators

##### 3.5.1.1 Specific outcome indicators

To examine the differential effects of RSH across various performance outcomes, subgroup analyses were conducted using outcome category as a moderator. The results showed that, within the domain of RSA, RSH produced significant moderate-to-large effects for RSA_Time [ES = 1.09, 95% CI (0.31, 1.86), *p* < 0.01] and RSA_N [ES = 1.00, 95% CI (0.09, 1.92), *p* < 0.05], suggesting that RSH can effectively improve both the total number and cumulative duration of maximal sprints. A moderate and significant effect was also observed for RSA_Sprint [ES = 0.82, 95% CI (0.08, 1.56), *p* < 0.05], indicating benefits in sprint speed during repeated efforts. In contrast, other RSA-related outcomes such as RSA_P (ES = 0.21, *p* = 0.51), RSA_DEC (ES = 0.56, *p* = 0.13), RSA_D (ES = 0.11, *p* = 0.82), and RSA_A (ES = 0.39, *p* = 0.18) showed positive trends but did not reach statistical significance. The effects for 10 m sprint and RSA_10 m were also nonsignificant with wide confidence intervals, indicating insufficient evidence at present.

**Figure 5:**
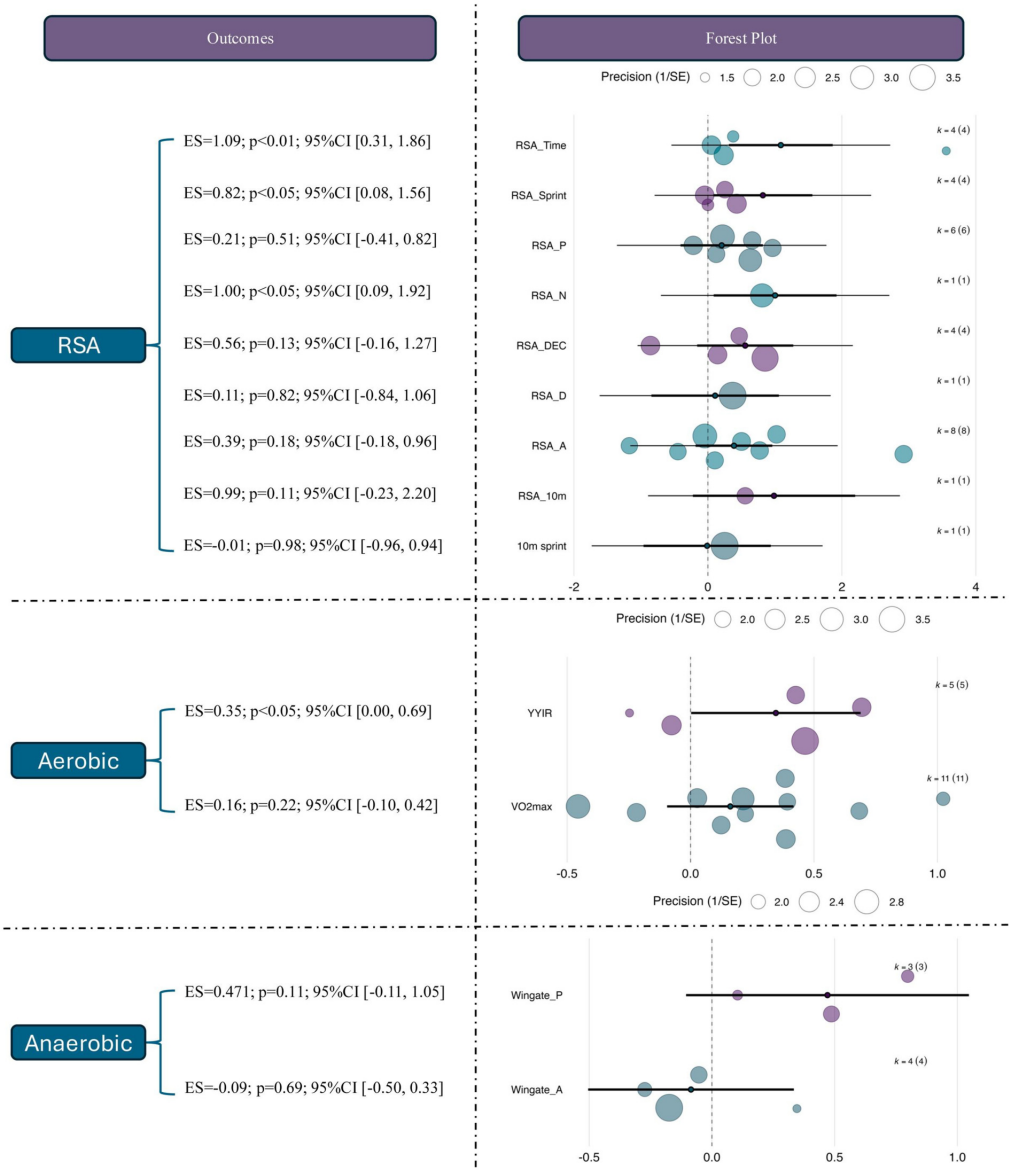
Effects of RSH on specific outcome measures across different performance domains. ES, effect size; CI, confidence interval; RSA, repeated sprint ability; RSA_Time, repeated sprint ability total time; RSA_Sprint, repeated sprint ability sprint time; RSA_N, repeated sprint ability number; RSA_P, repeated sprint ability peak; RSA_DEC, repeated sprint ability decay; RSA_D, repeated sprint ability distance; RSA_A, repeated sprint ability average; 10 m sprint, 10-meter sprint test; YYIR, Yo-Yo intermittent recovery test; VO2max, maximal oxygen uptake; Wingate_P, wingate peak power; 2ingate_A, Wingate average power.

In the aerobic capacity domain, RSH demonstrated a significant effect on the Yo-Yo Intermittent Recovery test [YYIR; ES = 0.35, 95% CI (0.00, 0.69), *p* < 0.05], indicating enhanced intermittent running endurance. However, the effect on VO_2max_ was not statistically significant [ES = 0.16, 95% CI (–0.10, 0.42), *p* = 0.22], possibly due to short intervention durations or insufficient training load. Regarding anaerobic performance, RSH showed a moderate but non-significant effect on Wingate peak power (ES = 0.47, *p* = 0.11) and a small, non-significant, and negative effect on average power (ES = –0.09, *p* = 0.69), suggesting that short-duration high-intensity power output may not be a primary adaptation target of RSH. See in Figure 5.

##### 3.5.1.2 Type of training modality

To explore the potential differences in adaptation across various exercise modalities, subgroup analyses were conducted using training mode (running, circuit-based training, or cycling-based training) as a categorical moderator. The results revealed that studies using rowing ergometer-based training observed the largest and statistically significant effect size [ES = 0.81, 95% CI (0.33, 1.29), *p* < 0.01], which was notably greater than those observed in running [ES = 0.31, 95% CI (0.10, 0.52), *p* < 0.01] and cycling interventions [ES = 0.26, 95% CI (0.00, 0.51), *p* = 0.05]. This suggests that under hypoxic conditions, rowing-based RSH may confer superior performance benefits, possibly due to its greater whole-body metabolic demand.

**Figure 6:**
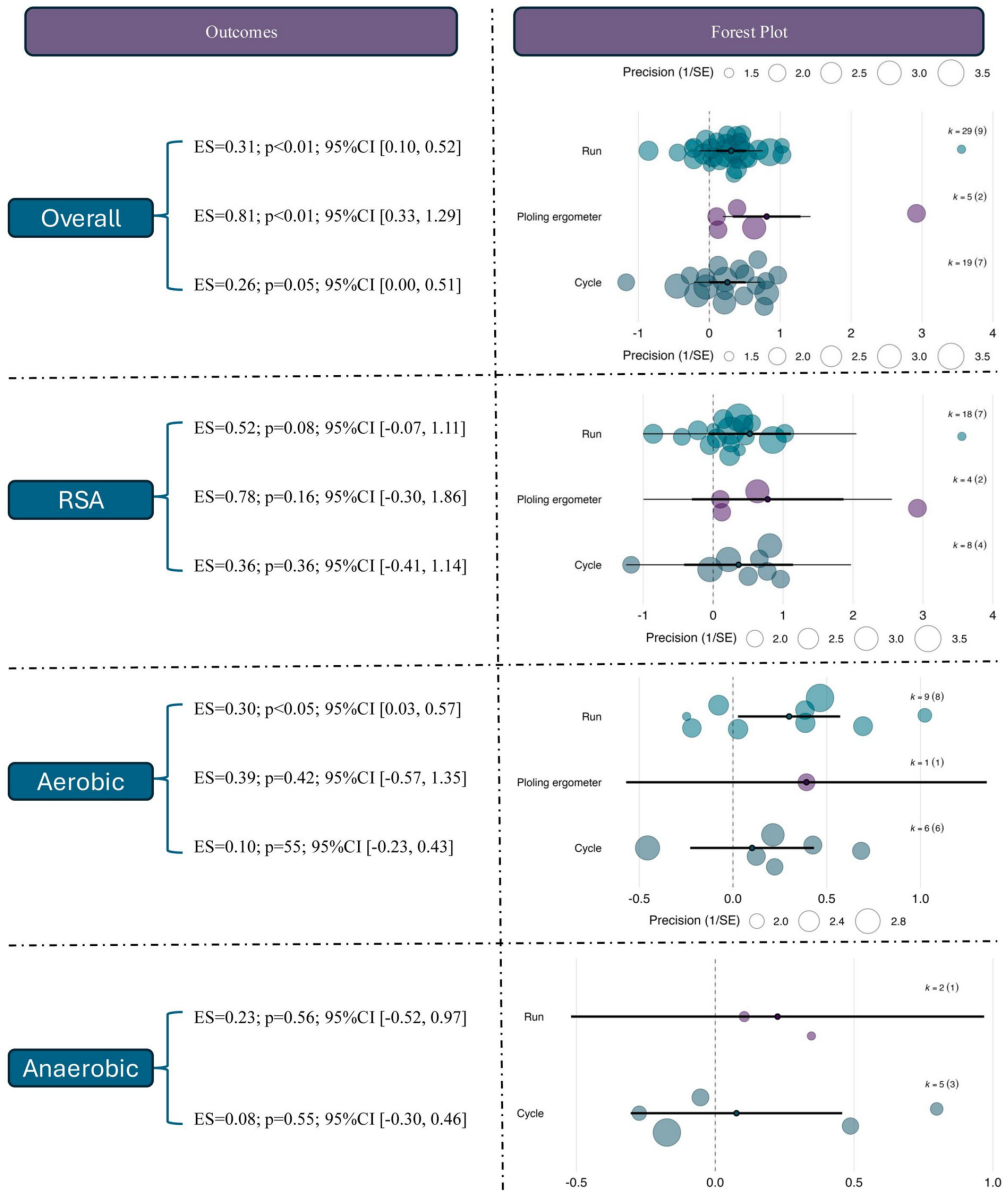
Effects of RSH on athletic performance across different intervention modalities.

For RSA outcomes, although none of the three training modalities yielded statistically significant results, rowing-based RSH showed the largest effect size [ES = 0.78, 95% CI (–0.30, 1.86), *p* = 0.16], followed by running (ES = 0.52, *p* = 0.08) and cycling (ES = 0.36, *p* = 0.36), suggesting a potential advantage of rowing-based RSH in enhancing sprint capacity, despite current evidence remaining inconclusive. Regarding aerobic capacity, only the running-based intervention reached statistical significance [ES = 0.30, 95% CI (0.03, 0.57), *p* < 0.05], while rowing (ES = 0.39, *p* = 0.42) and cycling (ES = 0.10, *p* = 0.55) showed non-significant positive trends with wide confidence intervals. For anaerobic outcomes, none of the modalities produced statistically significant effects. Effect sizes were ES = 0.23 for running and ES = 0.08 for cycling, suggesting that anaerobic performance may not be the most responsive endpoint to RSH interventions. See in Figure 6.

##### 3.5.1.3 Sex

The moderating role of sex in the effects of RSH was further examined. For overall performance outcomes, studies involving male participants showed a statistically significant moderate effect [ES = 0.45, 95% CI (0.25, 0.65), *p* < 0.01], whereas female-only (ES = 0.18, *p* = 0.50) and mixed-sex groups (ES = 0.12, *p* = 0.47) did not reach statistical significance. This suggests that current evidence primarily reflects RSH effects in male populations.

**Figure 7:**
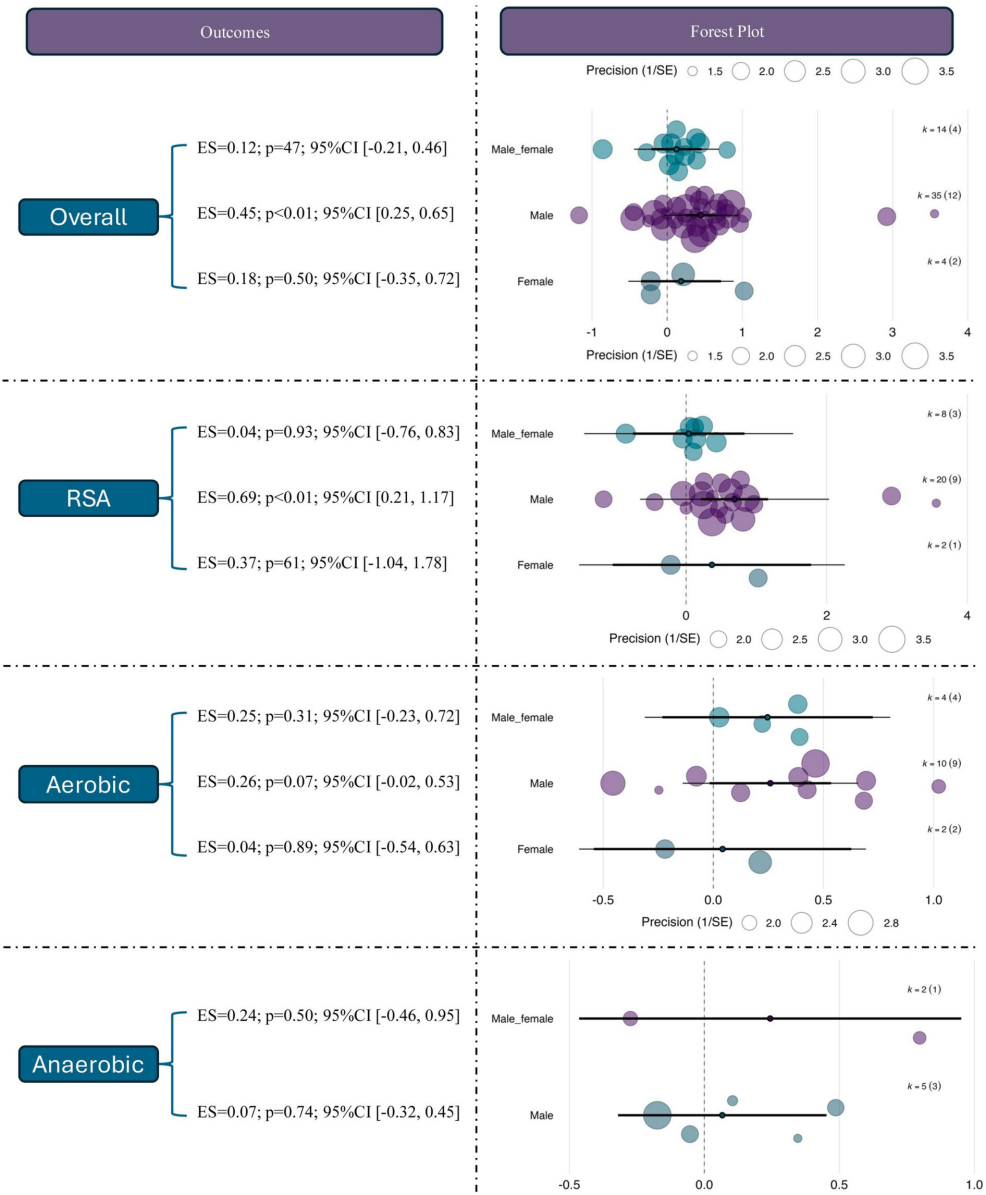
Effects of RSH on athletic performance stratified by sex.

For RSA outcomes, male participants demonstrated a significant improvement [ES = 0.69, 95% CI (0.21, 1.17), *p* < 0.01], while no significant effects were observed in female (ES = 0.37, *p* = 0.61) or mixed-sex groups (ES = –0.04, *p* = 0.93). Notably, the confidence interval for the female group was wide (95% CI [–1.04, 1.78]), indicating small sample sizes and result instability. In the aerobic domain, the male subgroup showed a near-significant trend (ES = 0.26, *p* = 0.07), while no effects were observed in the female (ES = –0.04) or mixed groups (ES = –0.25), both with *p* > 0.3. For anaerobic performance, none of the sex-based subgroups showed significant effects; the effect sizes were ES = –0.07 (*p* = 0.74) for males and ES = –0.24 (*p* = 0.50) for mixed-sex samples. See in Figure 7.

#### 3.5.2 Continuous moderators

##### 3.5.2.1 Meta-regression of continuous predictors on overall effects

To further investigate the moderating role of training parameters in RSH interventions, a multivariate meta-regression model was constructed based on 53 effect sizes. Four continuous variables were included: FiO_2_, ER, intervention duration (in weeks), and weekly training frequency (per). A multilevel meta-regression framework was applied to account for between-study heterogeneity.

The results showed that both exercise-to-rest ratio [Estimate = 0.393, SE = 0.182, 95% CI (0.037, 0.749), *p* = 0.0307] and intervention duration [Estimate = 0.457, SE = 0.212, 95% CI (0.041, 0.872), *p* = 0.0314] had significant positive predictive effects on training outcomes. These findings suggest that higher exercise-to-rest ratios and longer intervention periods are associated with enhanced effectiveness of RSH. See in Table 2.

**Table 2:** Meta-regression results of continuous moderators on overall effect sizes.

Moderator	ES	SE	95% CI	*p*
FiO_2_	3.836	1.988	(-0.061,7.732)	0.0537
Exercise-to-rest ratio (E:R)	0.393	0.182	(0.037,0.749)	0.0307
Intervention duration (weeks)	0.457	0.212	(0.041,0.872)	0.0314
Weekly training frequency	0.462	0.253	(−0.034,0.958)	0.0679

**Figure 8:**
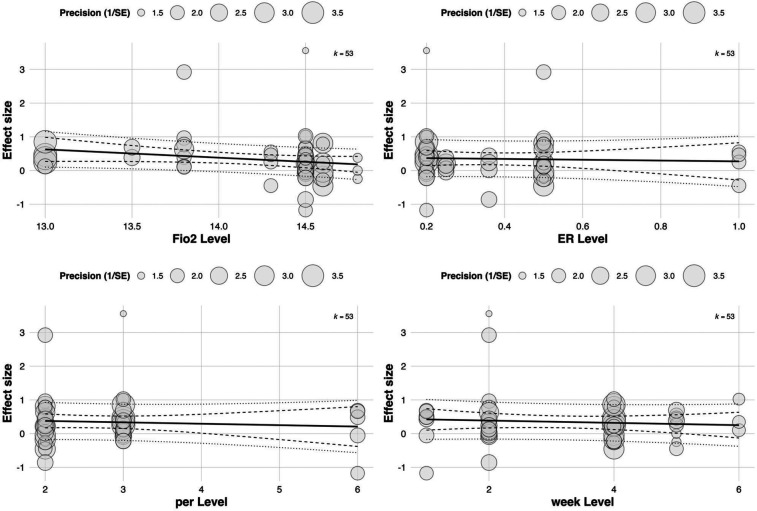
Meta-regression of continuous predictors on the overall effect of RSH on athletic performance.

Although FiO_2_ (Estimate = 3.836, *p* = 0.0537) and weekly training frequency (Estimate = 0.462, *p* = 0.0679) did not reach statistical significance, both showed positive trends. Notably, the relatively large regression coefficient for FiO_2_ indicates that even small variations in oxygen concentration may exert substantial influence on training effects, highlighting its potential value as a moderator. See in Figure 8.

##### 3.5.2.2 Meta-regression of continuous predictors on RSA effects

To further explore sources of heterogeneity in the effects of RSH on RSA, a multivariate meta-regression model was constructed including four continuous moderators: FiO_2_, exercise-to-rest ratio, intervention duration (in weeks), and weekly training frequency. The estimated regression coefficients for RSA effect sizes were as follows: FiO_2_ [Estimate = 3.345, SE = 5.814, 95% CI (–8.051, 14.740), *p* = 0.565], exercise-to-rest ratio [Estimate = 0.672, SE = 0.430, 95% CI (–0.171, 1.515), *p* = 0.118], intervention duration [Estimate = 0.734, SE = 0.478, 95% CI (–0.203, 1.671), *p* = 0.125], and training frequency [Estimate = 0.868, SE = 0.539, 95% CI (–0.188, 1.924), *p* = 0.107]. See in Table 3.

**Table 3:** Meta-regression results of continuous moderators on RSA effect sizes.

Moderator Variables/Regression Results	ES	SE	95% CI	*p*
FiO_2_	3.345	5.814	(−8.051,14.74)	0.565
Exercise-to-rest ratio (E:R)	0.672	0.43	(−0.171,1.515)	0.118
Intervention duration (weeks)	0.734	0.478	(−0.203,1.671)	0.125
Weekly training frequency	0.868	0.539	(−0.188,1.924)	0.107

**Figure 9:**
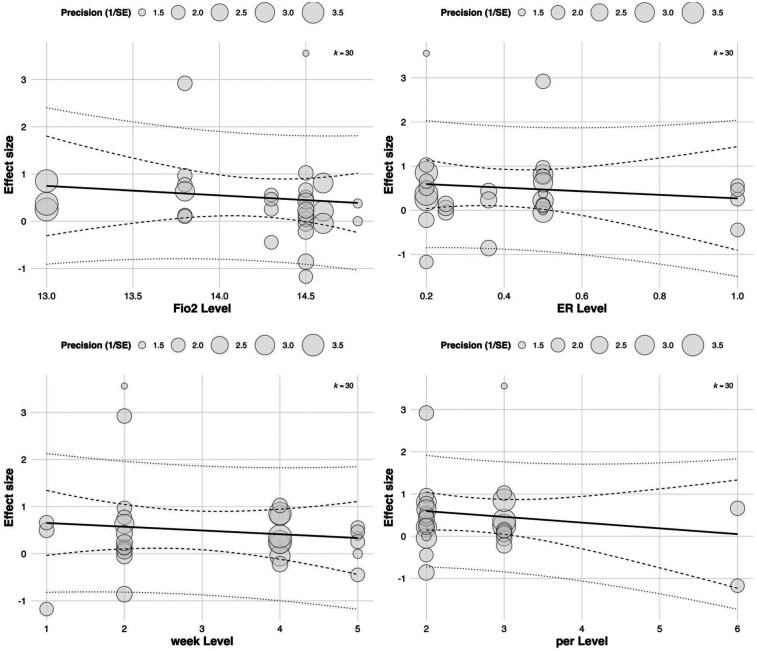
Meta-regression of continuous predictors on the effect of RSH on RSA.

Although all regression coefficients were positive—suggesting that increases in these parameters may theoretically enhance RSA outcomes—none reached statistical significance. These findings indicate that current evidence is insufficient to confirm the independent moderating effects of these training parameters on RSA improvement (all *p* > 0.05). See in Figure 9.

##### 3.5.2.3 Meta-regression of continuous predictors on aerobic capacity effects

In the subgroup analysis focusing on aerobic capacity outcomes, meta-regression was conducted to assess the influence of four continuous variables: FiO_2_, exercise-to-rest ratio, intervention duration, and weekly training frequency. The results showed a significant moderating effect of exercise-to-rest ratio [Estimate = 0.703, SE = 0.263, 95% CI (0.187, 1.220), *p* = 0.00758], suggesting that higher training intensity (i.e., shorter rest intervals) may be more beneficial for improving aerobic performance. The corresponding regression plot also indicated a clear upward trend in effect sizes with increasing exercise-to-rest ratios.

The regression coefficient for FiO_2_ was 3.583 [SE = 2.265, 95% CI (–0.857, 8.022), *p* = 0.114], which did not reach statistical significance. However, the regression plot revealed a slight downward trend in effect sizes with increasing FiO_2_ concentration, suggesting that excessively high hypoxic intensity may not confer additional aerobic benefits and that the level of oxygen pressure should be carefully calibrated. See in Table 4.

**Table 4:** Meta-regression results of continuous moderators on aerobic capacity effect sizes.

Moderator	ES	SE	95% CI	*p*
FiO_2_	3.583	2.265	(−0.857, 8.022)	0.114
Exercise-to-rest ratio (E:R)	0.703	0.263	(0.187, 1.22)	0.00,758
Intervention duration (weeks)	0.189	0.289	(−0.377, 0.755)	0.513
Weekly training frequency	−0.345	0.352	(−1.036, 0.346)	0.328

**Figure 10:**
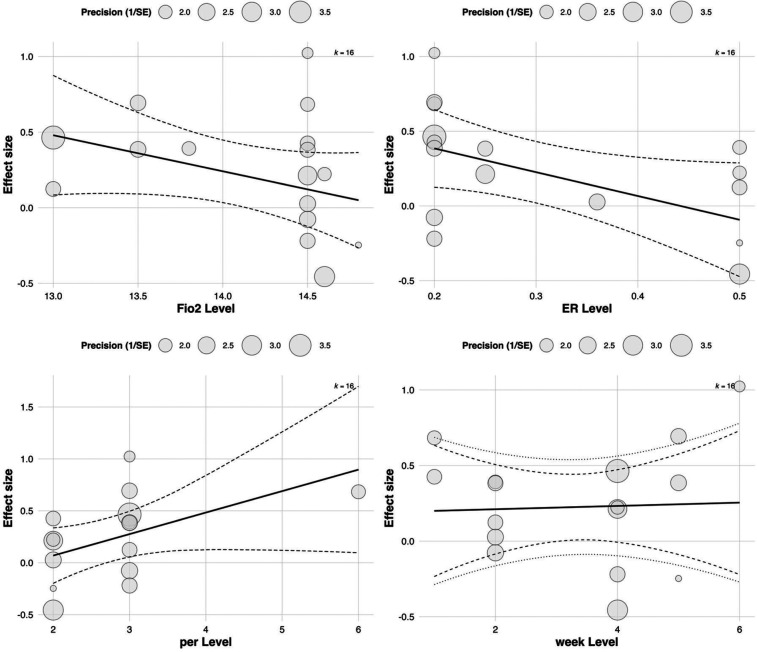
Meta-regression of continuous predictors on the effect of RSH on aerobic capacity.

Neither intervention duration (Estimate = 0.189, *p* = 0.513) nor weekly frequency (Estimate = –0.345, *p* = 0.328) showed statistically significant moderating effects. Notably, the plot indicated a negative correlation between training frequency and aerobic adaptation, potentially implying that excessively frequent training could induce fatigue or adaptive plateaus. These findings underscore the need for carefully periodized training strategies to optimize outcomes. See in Figure 10.

##### 3.5.2.4 Meta-regression of continuous predictors on anaerobic performance effects

In the multilevel meta-regression analysis of anaerobic performance outcomes, none of the continuous moderators demonstrated statistically significant effects. Specifically, the regression coefficient for FiO_2_ was ES = 30.72 with a standard error of 50.40 [95% CI (–68.07, 129.51), *p* = 0.542], indicating a non-significant relationship between oxygen concentration and anaerobic performance improvements. The regression coefficient for exercise-to-rest ratio was ES = 0.36 [SE = 0.45, 95% CI (–0.52, 1.24), *p* = 0.424], also lacking statistical significance. See in Table 5.

**Table 5:** Meta-regression results of continuous moderators on anaerobic performance effect sizes.

Moderator	ES	SE	95% CI	*p*
FiO_2_	30.718	50.404	(−68.072, 129.509)	0.542
Exercise-to-rest ratio (E: R)	0.358	0.448	(−0.52, 1.237)	0.424
Intervention duration (weeks)	0.149	0.397	(−0.629, 0.926)	0.708
Weekly training frequency	−0.05	0.377	(−0.789, 0.689)	0.895

**Figure 11:**
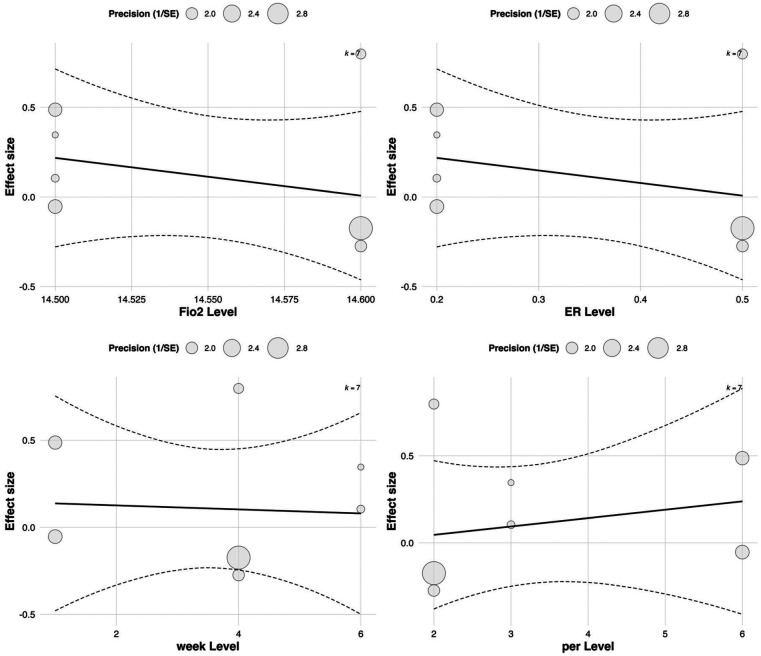
Meta-regression of continuous predictors on the effect of RSH on anaerobic performance.

Similarly, no significant effects were observed for intervention duration [ES = 0.15, SE = 0.40, 95% CI (–0.63, 0.93), *p* = 0.708] or training frequency [ES = –0.05, SE = 0.38, 95% CI (–0.79, 0.69), *p* = 0.895]. The corresponding bubble plots showed largely flat regression lines with wide confidence bands, further supporting the limited moderating role of these continuous variables in explaining the variability in anaerobic performance outcomes. See in Figure 11.

### 3.6 Publication bias assessment

To assess the robustness of the main model, a sensitivity analysis was first conducted by sequentially excluding each study and re-estimating the model. The results showed that no single study substantially altered the overall effect size, indicating high stability of the findings. Additionally, a PI was used to evaluate the generalizability of the results; the PI for the overall effect was (–0.16, 0.85), suggesting that despite some heterogeneity across studies, the estimated effect remains sufficiently robust and generalizable.

Publication bias was evaluated using contour-enhanced funnel plots combined with Egger's regression test. The funnel plots for the overall model and the RSA subgroup were largely symmetrical, with no obvious signs of asymmetry, indicating a low risk of publication bias. In the aerobic and anaerobic subgroups, the data points appeared slightly skewed; however, the Egger regression lines did not significantly deviate from the vertical axis, and no statistical significance was detected. Further adjustments using the trim-and-fill method revealed only minor changes in the overall effect estimates after imputing potentially missing studies, supporting the reliability of the original results. See in Figure 4.

## 4 Discussion

### 4.1 Effects of RSH on athletic performance

This study systematically evaluated the effects of repeated-sprint training in hypoxia (RSH) on multiple dimensions of athletic performance using a multilevel meta-analytic approach. The results demonstrated that RSH had a significant moderate effect on improving repeated sprint ability (RSA), a small but statistically significant advantage for sport-specific aerobic capacity, and no clear effect on anaerobic capacity due to substantial heterogeneity and inconsistent findings.

RSA reflects an athlete's ability to perform multiple sprints over a short period while maintaining sprint quality with limited recovery, and it is widely used to predict performance in team sports. The meta-analysis revealed a pooled effect size of ES = 0.50 [95% CI (0.12, 0.88), *p* < 0.01], representing the most prominent effect across all outcome types. Several original studies have confirmed the robustness of this finding. For instance, Faiss et al. (11) reported that RSH upregulated key molecular markers such as carbonic anhydrase and MCT transporters, enhancing lactate clearance and proton transport efficiency during sprints. Gatterer et al. (35) observed a significant reduction in RSA fatigue slope among adolescent football players, suggesting improved fatigue resistance. Brocherie et al. (30) further demonstrated that performance gains persisted for up to three weeks post-intervention in elite field hockey players, with total sprint time reduced by 3.5% (*p* < 0.01), indicating potential medium-term retention effects. On the other hand, some studies have expressed caution regarding RSH efficacy, especially when discrepancies exist between the training and testing modalities, such as running vs. cycling, linear vs. change-of-direction movement, or mismatched loading protocols (43, 44). These findings suggest that training–testing specificity is a critical moderator of RSH effectiveness.

Regarding aerobic capacity, the pooled effect size was ES = 0.23 [95% CI (0.02, 0.44), *p* = 0.03], representing a small but statistically significant effect. Pramkratok et al. (40) reported a 7.5% increase in VO_2max_ after six weeks of RSH, along with significant upregulation of the HIF-1α and VEGF pathways, suggesting that the aerobic benefits of RSH may be mediated by hypoxia-induced angiogenesis and mitochondrial biogenesis. However, Kasai et al. (38) and Brocherie et al. (14) failed to observe meaningful changes in _VO2max_ among elite athletes, and the pooled confidence interval crossed the null, indicating that VO_2max_ responses may depend on training dose, baseline fitness, and measurement sensitivity. It is noteworthy that RSH produced more consistent improvements in sport-specific aerobic indicators such as the Yo-Yo Intermittent Recovery (YYIR) test. For example, Galvin et al. (34) and Hamlin et al. (37) both reported superior improvements in YYIR1 performance in the RSH group compared with RSN, possibly due to enhanced activation of peripheral metabolic pathways (e.g., muscle oxygen uptake and lactate clearance) rather than central cardiovascular adaptations. This suggests that the aerobic improvements from RSH may reflect “specific adaptation” rather than “maximal physiological enhancement.”.

For anaerobic capacity, the meta-analysis did not reveal statistically significant effects [ES = 0.11, 95% CI (–0.23, 0.45), *p* = 0.67]. While some studies, such as Gutknecht et al. (45) and Woorons et al. (46), reported increases in Wingate peak and mean power (+11.9% and +6.2%, respectively, both *p* < 0.05), most studies found no systematic advantage of RSH over RSN. For instance, Kasai et al. (38) and Gatterer et al. (47) reported no significant between-group differences in peak or mean power output. Potential explanations include insufficient stimulation of anaerobic pathways by short-duration training and the limited sensitivity of the Wingate test in detecting subtle metabolic adaptations.

In summary, RSH exerts a consistent and significant positive effect on RSA and shows modest benefits for sport-specific aerobic performance, particularly in high-intensity intermittent sports. However, its impact on VO_2max_ and anaerobic capacity appears limited and may depend on a combination of training dose, modality, and individual baseline characteristics.

### 4.2 Moderating variables

To further explore how study characteristics influence the effects of RSH, a series of categorical and continuous moderators were tested using a multilevel meta-regression framework. The results showed that these moderators played an important role in explaining between-study heterogeneity, suggesting that the efficacy of RSH may be shaped by training dose parameters, physiological background, and implementation strategies. Among the categorical variables, outcome type emerged as a significant moderator. RSH produced the largest and most consistent effects on RSA outcomes [e.g., sprint count, fatigue index; ES = 0.50, 95% CI (0.12, 0.88), *p* < 0.01], whereas the effects on VO2max (ES = 0.23, *p* = 0.67) and anaerobic performance (ES = 0.11, *p* = 0.67) were less stable. These findings suggest that RSH may be more suited for improving intermittent performance rather than eliciting substantial gains in cardiorespiratory endurance or anaerobic power output. Training modality also significantly moderated the effects, with running-based RSH interventions yielding greater benefits than cycling or rowing ergometer protocols. This may be due to training–testing specificity: the closer the match between training and testing modalities, the more pronounced the adaptation (48).

Subgroup analysis by sex revealed no statistically significant differences between groups; however, most included studies predominantly involved male athletes, which inherently limits the generalizability of sex-based findings. Although slightly larger effect sizes were observed in mixed-sex or female subgroups, these results should be interpreted with caution due to the small number of female-specific studies and limited statistical power. This gender imbalance underscores a critical gap in the current RSH literature and highlights the need for future trials to systematically examine sex-specific responses to hypoxic sprint training. Among the continuous moderators, inspired oxygen fraction (FiO₂), exercise-to-rest ratio (E:R), intervention duration (in weeks), and weekly training frequency were examined within a multivariate regression framework. Among these, FiO₂ demonstrated a marginally significant association with training outcomes (ES = 3.84, SE = 1.99, *p* = 0.0537), suggesting a potential—but not conclusive—moderating role. Given the proximity of this *p*-value to the conventional significance threshold, caution is warranted in interpreting this result as evidence of a reliable effect. Rather than inferring causality, these findings should be viewed as hypothesis-generating and indicative of trends that merit further investigation. Notably, prior literature has suggested a nonlinear dose–response relationship between inspired oxygen levels and performance outcomes (49, 50). Within this context, moderate hypoxic exposures (FiO₂ = 14.0%–14.5%) are often hypothesized to balance sufficient hypoxic stress with tolerable physiological demand. However, the present data do not permit firm conclusions regarding an optimal FiO₂ range. Levels below 14% have been associated in some studies with central nervous system suppression and impaired motor output, while more severe hypoxia (FiO₂ ≤ 12%) may exacerbate metabolic stress without concomitant performance benefits. These insights, although mechanistically plausible, remain speculative in the absence of statistically robust moderator effects and should be interpreted with due methodological restraint.

Regarding the exercise-to-rest ratio, results suggested that relatively longer rest intervals (i.e., lower E: *R* values) may enhance power output and metabolic recovery (ES = 0.393, *p* = 0.0307), a finding consistent with studies by Raberin et al. (51, 52) and Faiss et al. (3). Longer inter-set recovery may promote phosphocreatine resynthesis and oxygenation, facilitating peripheral adaptation and improving muscular oxygen delivery. Additionally, it has been shown that nitric oxide production and vasodilation are enhanced under hypoxic conditions, particularly in “all-out sprint + incomplete recovery” protocols, where RSH may stimulate greater muscle reperfusion and fast-twitch fiber recruitment (11, 53).

Training frequency and intervention duration also showed marginally significant trends. In endurance-related outcomes such as VO_2max_, a nonlinear pattern was observed, where longer interventions (≥4 weeks) and moderate weekly frequency (3–4 sessions/week) were more effective for promoting mitochondrial biogenesis and metabolic remodeling (40, 54). However, no consistent patterns were found for anaerobic or explosive performance outcomes.

It is worth noting that baseline training status may also influence RSH outcomes. Participants with lower training backgrounds (e.g., sedentary females or recreationally active youth) often exhibit greater adaptive potential in VO_2max_ and aerobic capacity (55, 56), whereas elite athletes may require more potent stimuli to elicit further adaptations due to approaching physiological ceilings (57). Although sex did not significantly moderate overall effects, minor fluctuations in female performance may occur across menstrual cycle phases. Nonetheless, existing studies support the safety and adaptability of RSH across both sexes (38, 58).

In summary, the training effects of RSH are influenced by multiple interacting factors. Optimal adaptations appear to be achieved under moderate hypoxic doses (FiO_2_ = 14%), with a balanced exercise-to-rest ratio, intervention durations of at least four weeks, and individualized training frequency.

### 4.3 Potential mechanisms

Repeated-sprint training in hypoxia (RSH) induces specific physiological adaptations by combining maximal sprint bouts with incomplete recovery under reduced oxygen conditions. Its efficacy is underpinned by three key mechanisms: improved oxygen delivery, enhanced phosphagen system function, and glycolytic remodeling.

First, the combined hypoxic and high-intensity load preferentially recruits fast-twitch (FT) muscle fibers, which are particularly sensitive to oxygen availability. RSH promotes vasodilation via nitric oxide (NO) signaling, enhancing perfusion and oxygen uptake in FT-dominant regions, thereby improving sprint capacity (59). This response is mediated by shear stress-induced activation of endothelial and neuronal NO synthase (60). Second, RSH enhances energy supply by accelerating phosphocreatine (PCr) resynthesis and increasing PCr and glycogen stores, enabling sustained ATP regeneration (61). Short-term interventions have demonstrated 14%–18% increases in glycogen and 21% in PCr after RSH (39). In high-intensity, short-rest settings, PCr recovery is more strongly associated with sprint performance than acid–base buffering (62). Third, RSH activates hypoxia-inducible factor-1α (HIF-1α), which upregulates glycolytic and buffering proteins such as monocarboxylate transporter 4 (MCT4) and carbonic anhydrase III (CA3), improving lactate clearance and pH stability (63). RSH enhances MCT4 expression in FT fibers (±20%) and increases CA3 activity more than normoxic training (11, 64). Conversely, mitochondrial oxidative phosphorylation markers (e.g., COX IV, SDHB) are unchanged or downregulated (65), indicating a shift toward glycolytic metabolism.

Together, these mechanisms enhance repeated-sprint ability by improving FT fiber oxygenation, anaerobic energy regeneration, and metabolic resilience under hypoxic stress.

### 4.4 Practical recommendations for intervention design

Given the diverse physiological demands across sports, RSH protocols should be tailored to specific athletic contexts to enhance transferability and training efficacy. For team sports (e.g., football, rugby), where repeated sprint ability (RSA) and recovery under fatigue are critical, sessions should include 2–3 weekly bouts of 5–10 s maximal sprints with short recovery intervals (1:2–1:4), under moderate hypoxia (FiO₂ = 14.0%–14.5%). Sport-specific drills such as shuttle runs or change-of-direction sprints are recommended to maximize ecological validity. For individual sprint or power athletes (e.g., track sprinters, cyclists), emphasis should be placed on neuromuscular quality. Use fewer repetitions with full recovery between sets (e.g., 3–5 min), gradually progressing from FiO₂ 15.5%–14.0% to elevate metabolic load without impairing technique. For endurance athletes (e.g., rowers, middle-distance runners), RSH may complement aerobic base training by improving buffering capacity. Sessions can include 20–30 s efforts at near-maximal intensity, with incomplete recovery and FiO₂ between 13.5%–14.5%. Across all sports, careful monitoring of training load via heart rate variability, RPE, and oxygen saturation is advised to avoid non-functional overreaching. RSH should be periodized within mesocycles and supported with appropriate recovery strategies (e.g., sleep, nutrition). These targeted guidelines provide a practical framework for integrating RSH across diverse training environments, maximizing its applied value in sport-specific performance enhancement.

### 4.5 Research limitations

Despite the overall robustness of the present meta-analysis, several limitations warrant consideration. First, although funnel plot symmetry and Egger's test suggested a low risk of publication bias, subtle small-study effects or selective reporting cannot be entirely ruled out—especially in outcome domains with fewer effect sizes, such as anaerobic performance or female-only samples. The concentration of studies within a few research groups may also introduce potential investigator or institutional bias. Second, most included interventions were of short duration (1–6 weeks), limiting the ability to assess whether observed gains in performance reflect transient physiological responses or sustainable adaptations. Long-term effects on training retention, detraining resistance, and competition-season performance remain unknown. Third, the lack of follow-up assessments precluded insight into the persistence of RSH-induced adaptations. Although moderate hypoxic doses (∼14.0% FiO₂) and adequate training duration (≥4 weeks) appeared beneficial, current evidence offers limited understanding of the dose–response dynamics or potential maladaptations associated with chronic hypoxic exposure. These limitations highlight the need for well-powered, longitudinal trials incorporating extended follow-up, diverse populations, and rigorous reporting standards. Future research should aim to clarify optimal hypoxic dosing strategies, characterize individualized response patterns, and evaluate the long-term efficacy and safety of RSH in both elite and sub-elite athletic settings.

## 5 Conclusion

This systematic review and multilevel meta-analysis comprehensively evaluated the effects of RSH on athletic performance. The findings indicate that RSH can significantly enhance overall performance, with a clear advantage over normoxic training particularly in improving RSA. Moderate-to-small effects were observed for aerobic and anaerobic outcomes, accompanied by considerable heterogeneity. Moderator analyses revealed that training effects varied significantly across sport modalities, sex composition, and outcome types. More pronounced benefits were observed when training and testing modalities matched (e.g., running-based protocols), in predominantly male samples, and when specific outcome measures such as RSA-Time and YYIR were employed. Among continuous moderators, higher exercise-to-rest ratios and moderate intervention durations were significant positive predictors of training efficacy, underscoring the importance of scientifically informed parameter design to maximize training gains. From a mechanistic perspective, RSH offers distinct advantages over normoxic training due to its ability to simultaneously activate multiple adaptation pathways under hypoxia–high-intensity coupling. These include intensity-dependent vasodilation mediated by nitric oxide (NO), optimized phosphocreatine (PCr) resynthesis kinetics, and upregulation of glycolytic and buffering systems driven by hypoxia-inducible factor 1-alpha (HIF-1*α*).

Taken together, RSH represents a physiologically targeted training modality that is particularly suitable for competitive sports requiring repeated high-intensity efforts. However, its effectiveness is moderated by several factors, including oxygen pressure, training structure, individual fitness level, and training status. Future research should aim to refine optimal dosing strategies, clarify the mechanisms underlying improvements in different performance domains, and characterize individualized response patterns. Expanding the application of RSH to female athletes, youth populations, and varying levels of athletic expertise will also be essential to advance the precision and efficiency of hypoxia-based training interventions.

## Data Availability

The original contributions presented in the study are included in the article/Supplementary Material, further inquiries can be directed to the corresponding author.
