# Correction: A multilevel meta-analysis of the effects of repeated sprint training in hypoxia on athletic performance

**DOI:** 10.3389/fspor.2026.1780346

**Published:** 2026-02-02

**Authors:** Meng Han, Binglin Liu

**Affiliations:** 1 The College of Tianjin Renai, Tianjin, China; 2 College of Sports Training Science, Tianjin University of Sport, Tianjin, China

**Keywords:** repeated-sprint training, hypoxia, athletic performance, meta-analysis, repeated-sprint ability, aerobic capacity, anaerobic power, training adaptation

Affiliation The College of Tianjin Renai, Tianjin, China was omitted for author Meng Han. This affiliation has now been added for author Meng Han.

Author Meng Han was erroneously assigned to affiliation Department of Physical Education, Tianjin University of Sport, Tianjin, China. This affiliation has now been removed for author Meng Han.

Affiliation College of Sports Training Science, Tianjin University of Sport, Tianjin, China was erroneously given as Department of Physical Education, Tianjin University of Sport, Tianjin, China.

The funding project “A Study on the Path to Enhance College Students' Physical Health Level Based on Functional Training”, grant number FZ2E2001, was erroneously omitted. The correct Funding statement reads:

“The author(s) declare that financial support was received for the research and/or publication of this article. FZ232001, Research on the path to improve the physical health level of college students based on functional training. The manuscript is supported by the project entitled ”A Study on the Path to Enhance College Students' Physical Health Level Based on Functional Training“ (Grant No.: FZ2E2001).”

The original version of this article has been updated.

